# The influence of pre-existing hypertension on coronavirus disease 2019 patients

**DOI:** 10.1017/S0950268820003118

**Published:** 2021-01-05

**Authors:** Zhi-Yao Wei, Rui Qiao, Jian Chen, Ji Huang, Hui Wu, Wen-Jun Wang, Hua Yu, Jing Xu, Chao Wang, Chong-Huai Gu, Hong-Jiang Li, Mi Li, Cong Liu, Jun Yang, Yang Wang, Hao-Yu Wang, Hai-Yan Qian, Yong-Jian Geng

**Affiliations:** 1 Department of Cardiology, Center for Coronary Heart Disease, Fu Wai Hospital, National Center for Cardiovascular Diseases of China, State Key Laboratory of Cardiovascular Disease, Chinese Academy of Medical Sciences and Peking Union Medical College, Beijing, China; 2 Department of Cardiology, Anqing Hospital affiliated to Anhui Medical University, Anqing City, Anhui Province, China; 3 Department of Cardiovascular Medicine, Guangdong Provincial Key Laboratory of Biomedical Imaging, Fifth Affiliated Hospital of Sun Yat-sen University, Zhuhai City, Guangdong Province, China; 4 Department of Cardiology, Beijing Anzhen Hospital, Capital Medical University, Beijing, China; 5 Department of Cardiology, Institute of Cardiovascular Disease, Yichang Central People's Hospital, China Three Gorges University, Yichang City, Hubei Province, China; 6 Department of Cardiology, Daye Chinese Medicine Hospital, Daye City, Hubei Province, China; 7 Department of Cardiology; the First Affiliated Hospital of USTC, Division of Life Sciences and Medicine, University of Science and Technology of China, Hefei City, Anhui Province, China; 8 Department of Infectious Diseases; the First Affiliated Hospital of USTC, Division of Life Sciences and Medicine, University of Science and Technology of China, Hefei City, Anhui Province, China; 9 Coronary Care Unit, Baoding No. 1 Central Hospital, Baoding City, Hebei Province, China; 10 Sixth Department of Hepatopathy, Baoding People's Hospital, Baoding City, Hebei Province, China; 11 Department of Gastroenterology, Yingcheng Chinese Medicine Hospital, Yingcheng City, Hubei Province, China; 12 Department of Otolaryngology, Daye People's Hospital, Daye City, Hubei Province, China; 13 Medical Research & Biometrics Center; Fu Wai Hospital, National Center for Cardiovascular Diseases of China, State Key Laboratory of Cardiovascular Disease, Chinese Academy of Medical Sciences and Peking Union Medical College, Beijing, China; 14 Division of Cardiology, Department of Internal Medicine, The Center for Cardiovascular Biology and Atherosclerosis Research, McGovern Medical School, University of Texas Health Science Center at Houston, Houston, Texas, USA

**Keywords:** COVID-19, prognosis, SARS-CoV-2

## Abstract

Hypertension represents one of the most common pre-existing conditions and comorbidities in Coronavirus disease 2019 (COVID-19) patients. To explore whether hypertension serves as a risk factor for disease severity, a multi-centre, retrospective study was conducted in COVID-19 patients. A total of 498 consecutively hospitalised patients with lab-confirmed COVID-19 in China were enrolled in this cohort. Using logistic regression, we assessed the association between hypertension and the likelihood of severe illness with adjustment for confounders. We observed that more than 16% of the enrolled patients exhibited pre-existing hypertension on admission. More severe COVID-19 cases occurred in individuals with hypertension than those without hypertension (21% *vs.* 10%, *P* = 0.007). Hypertension associated with the increased risk of severe illness, which was not modified by other demographic factors, such as age, sex, hospital geological location and blood pressure levels on admission. More attention and treatment should be offered to patients with underlying hypertension, who usually are older, have more comorbidities and more susceptible to cardiac complications.

## Introduction

Coronavirus disease 2019 (COVID-19) emerged in December 2019 and has triggered a health crisis in the world [1]. COVID-19 is caused by severe acute respiratory syndrome coronavirus 2 (SARS-CoV-2) [2], which infects host cells by receptor-mediated endocytosis in association with angiotensin-converting enzyme II (ACE2) [3]. The cruel case-fatality rate of COVID-19 is about 2.3%, while the rate for patients with underlying hypertension is 6.0%, according to an epidemiological study enrolling 44 672 confirmed cases [4].

The global prevalence of hypertension was estimated to be 1.13 billion in 2015 [5], and aggravated with advancing age, with a prevalence of 53.3% in people aged 50 years and older *vs.* 26.2% in those younger [6]. In the COVID-19 outbreak, hypertension is the most common comorbidities among COVID-19 patients, with the rate reported varying from 8.0% to 31.2% [7–10]. Owing to the high prevalence of hypertension, the relation between underlying hypertension and COVID-19 outcomes is of great concern to public health. A meta-analysis revealed that the incidence of hypertension was two-fold higher in severe cases than in their non-severe counterparts [9]. The proportion of hypertension in 406 deceased patients with COVID-19 infections was 39.7%, much higher than that in the overall population (12.6%) [11], although this analysis was not adjusted for other clinical features. Above all, pre-existing hypertension widely exists in COVID-19 patients and is more common in patients with severe conditions. However, whether hypertension was an independent risk factor for severe illness among COVID-19 patients remains controversial.

The present multi-centre, retrospective study aimed to explore the clinical characteristics of hospitalised COVID-19 patients with pre-existing hypertension, and to clarify the association between pre-existing hypertension and severe COVID-19.

## Methods

### Study design, participants and data recording

We conducted a multi-centred, retrospective study of laboratory-confirmed COVID-19 patients in six hospitals, respectively, in the eastern, southern, northern and central regions of China, which uniformly distributed in populated areas and geologically outside Wuhan city, including two teaching (enrolling 117 patients) and four non-teaching hospitals (enrolling 329 patients). A total of 498 consecutive patients with laboratory-confirmed COVID-19 admitted to these six hospitals from 3 January to 26 February 2020, were enrolled and retrospectively analysed. All patients were diagnosed as COVID-19, according to the guidelines of the World Health Organization and the National Health Commission of China [12, 13]. The clinical outcomes were monitored up to 8 March 2020, the final date of the last discharge. The ethics commissions of six participating hospitals have approved this study. Informed consent was waived as a retrospective study, and data analysis were performed anonymously. Given the retrospective nature of our study, patients or the public were not involved in the design, or conduct, or reporting or dissemination plans of our research.

The demographic, clinical information and therapeutic procedures for all the included COVID-19 patients were collected by experienced local clinicians, and entered into a computerised database and cross-checked. If any core data were missing, requests for clarification were sent to local coordinators who then directly contacted the attending clinicians.

### Definitions

Hypertension was defined as systolic blood pressure over 140 mmHg or diastolic blood pressure over 90 mmHg or self-reported use of an antihypertensive drug in the past two weeks. Pre-existing hypertension was identified based on documented admission notes. Fever was defined as an axillary temperature of 37.3 °C or higher. We identified patients with severe COVID-19, which was defined as admission to the intensive care unit (ICU), the use of invasive or non-invasive mechanical ventilation or death.

### Statistical analyses

Descriptive data were expressed as mean ± standard deviation (s.d.) or median with quartiles for continuous variables and number (percentage) for categorical variables. Normal distribution of continuous variables was assessed with the Shapiro–Wilk test and Q–Q plot. Differences between patients with pre-existing hypertension and those without were assessed with two-sample *t*-test or Wilcoxon rank-sum test for continuous variables and *χ*^2^ or Fisher's exact test for categorical variables.

Multivariate logistic regression analyses were performed to assess the adjusted odds ratio (OR) of patients with hypertension *vs.* those without hypertension on the severe COVID-19. Covariates that were significant in univariate analysis (*P* < 0.1) and with clear clinical significance were included in adjustment (age, sex, diabetes and coronary heart disease). Stratification for age (≤65 years *vs*. >65 years), sex (female *vs.* male), type of hospital (teaching *vs.* non-teaching) and blood pressure level on admission (>140/90 mmHg *vs.* ≤140/90 mmHg) were performed as secondary analysis. Exact logistic regression was leveraged in stratified analysis, given the limited sample size.

To verify the robustness of our findings, we conducted the first sensitivity analysis using a loose diagnostic criterion of hypertension. In this criterion, patients with a clear record of pre-existing hypertension or blood pressure over 140/90 mmHg on admission were regarded as hypertension. Then we performed the second sensitivity analysis based on the propensity score-based matching cohort. Propensity scores were calculated using a logistic regression model controlling for demographic data and confounding factors, including age, sex, hospital level, diabetes, hyperlipidaemia, chronic obstructive pulmonary disease, chronic kidney disease and coronary heart disease. COVID-19 patients with hypertension were matched 1-to-1 to those without hypertension using the nearest-neighbour strategy without replacement with a caliper of 0.1. The postmatch balance between the groups was assessed by standardised mean differences for which a difference of less than 10% for a given covariate indicates a relatively small imbalance.

Tests were two-sided with significance set at *α* less than 0.05. SPSS for Windows (Version 22.0, IBM), R software (Version 3.6.2, R Foundation for Statistical Computing) and SAS 9.4 system (SAS Institute, Cary, NC, USA) were used for statistical analysis.

## Results

### Demographic and clinical characteristics of COVID-19 patients with pre-existing hypertension

**Fig. 1.**
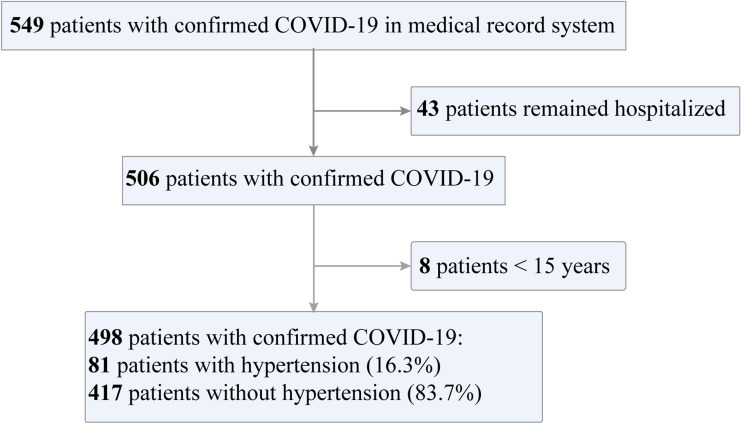
Flowchart of patient recruitment.

The current study enrolled 549 patients with laboratory-confirmed COVID-19 who were admitted to six hospitals as of 8 March 2020. Forty-three patients who remained hospitalised and 8 patients aged under 15 years were excluded. Figure 1 shows a flowchart for patient recruitment. Finally, the study population included 498 patients with confirmed COVID-19, including 81 (16.3%) of them had pre-existing hypertension. The median age of the 498 patients was 49.0 (37.0–59.0) years, and 239 (48.0%) of them were female. Generally, the hypertension patients were older than patients without hypertension (57.0 (51–68.5) years *vs.* 47.0 (36.0–57.0) years, *P* < 0.001) (Table 1). Pre-existing diabetes (19/81 (23.5%) *vs.* 24/416 (5.8%), *P* < 0.001), hyperlipidaemia (8/81 (9.8%) *vs.* 6/416 (1.4%), *P* = 0.001), cerebrovascular disease (7/81 (8.6%) *vs.* 2/416 (0.5%), *P* < 0.001), chronic kidney disease (4/81 (4.9%) *vs.* 2/416 (0.5%), *P* = 0.008) and chronic obstructive pulmonary disease (3/81 (3.7%) *vs.* 2/416 (0.5%), *P* = 0.032) had the higher frequency in hypertension patients than non-hypertension patients.

**Table 1.** Demographic and clinical characteristics of COVID-19 patients with or without hypertension

	Total (*n* = 498)	Hypertension (*n* = 81)	Non-hypertension (*n* = 416)	*P* value^a^
Age (IQR), years	49.0 (37.0–59.0)	57.0 (51–68.5)	47.0 (36.0–57.0)	<0.001
≥65	78 (15.7%)	29 (35.8%)	49 (11.8%)	<0.001
Female sex	239 (48.0%)	37 (45.7%)	202 (48.4%)	0.649
Comorbidities				
Diabetes	43 (8.6%)	19 (23.5%)	24 (5.8%)	<0.001
Hyperlipidaemia	14 (2.8%)	8 (9.8%)	6 (1.4%)	0.001
Coronary heart disease	10 (2.0%)	4 (4.9%)	6/425 (1.4%)	0.063
Chronic liver disease	10 (2.0%)	2 (2.5%)	8 (1.9%)	0.670
Cerebrovascular disease	9 (1.8%)	7 (8.6%)	2 (0.5%)	<0.001
Malignant tumour	8 (1.6%)	3 (3.7%)	5 (1.2%)	0.126
Chronic kidney disease	6 (1.2%)	4 (4.9%)	2 (0.5%)	0.008
Chronic obstructive pulmonary disease	5 (1.0%)	3 (3.7%)	2 (0.5%)	0.032
Signs and symptoms				
Fever	416 (83.5%)	68 (82.9%)	348 (83.7%)	0.828
Cough	367 (73.7%)	61 (75.3%)	306 (73.4%)	0.718
Fatigue	122 (24.5%)	22 (27.2%)	100 (24.0%)	0.543
Chest tightness	87 (17.5%)	23 (28.4%)	64 (15.3%)	0.005
Myalgia	58 (11.6%)	13 (16.0%)	45 (10.8%)	0.177
Abdominal discomfort/ diarrhoea/vomiting	55 (11.0%)	13 (16.0%)	42 (10.1%)	0.116
Pulse (s.d.), beats/min	85.3 ± 13.3	83.6 ± 13.9	85.6 ± 13.1	0.928
Systolic blood pressure (s.d.), mmHg^b^	126.2 ± 15.9	134.3 ± 20.6	124.6 ± 14.3	<0.001
Diastolic blood pressure (s.d.), mmHg^b^	78.6 ± 11.8	81.5 ± 14.8	78.1 ± 11.0	0.052

s.d., standard deviation.

a Significant difference (*P* < 0.05) was determined between the hypertension and non-hypertension subgroups.

b Blood pressure on admission was available in 492 patients.

The major symptoms on admission were fever (83.5%), cough (73.7%) and fatigue (24.5%), similar to previous reports. Hypertension patients were observed with a higher incidence of chest tightness than patients without hypertension (23/81 (28.4%) *vs.* 64/416 (15.3%), *P* = 0.005). Systolic blood pressure on admission was higher in patients with hypertension than those without hypertension (134.3 ± 20.6 mmHg *vs.* 124.6 ± 14.3 mmHg, *P* < 0.001), while diastolic blood pressure did not show a significant difference. It was observed that in 97 (19.5%) patients whose blood pressure on admission was over 140/90 mmHg, 67 (69.1%) of them did not have a known history of hypertension.

White blood cell count and neutrophil count were both higher in hypertension patients (5.5 (4.5–6.7) × 10⁹/l *vs.* 4.9 (3.9–5.8) × 10⁹/l, *P* = 0.001; 4.1(2.8–5.3) × 10⁹/l *vs.* 3.0 (2.2–4.2) × 10⁹/l, *P* < 0.001). C-reactive protein (31.1 (8.4–63.9) mg/l *vs.* 17.4 (5.0–36.7) mg/l, *P* = 0.001) was also higher, but the difference for interleukin-6 (6.5 (1.5–30.9) pg/ml *vs.* 4.4 (1.5–6.5) pg/ml, *P* = 0.058) did not reach a statistical significance. Elevated troponinI/T (>99th percentile upper reference limit, 19.4% *vs.* 9.9%, *P* = 0.032) were more common in hypertension patients compared with those without hypertension, along with higher levels of lactate dehydrogenase (232.0 (162.0–287.0) U/l *vs.* 189.0 (153.0–250.0) U/l, *P* = 0.003) and N-terminal pro-brain natriuretic peptide (152.0 (57.3–1151.4) pg/ml *vs.* 50.0 (23.2–193.0) pg/ml, *P* = 0.001), revealing more frequently occurred myocardial injury and dysfunction in hypertension patients (Table 2).

**Table 2.** Laboratory findings of COVID-19 patients with or without hypertension

	Total (*n* = 498)	Hypertension (*n* = 81)	Non-hypertension (*n* = 417)	*P* value^a^
White blood cells (IQR), × 10⁹/l	5.0 (4.0–5.9)	5.5 (4.5–6.7)	4.9 (3.9–5.8)	0.001
Neutrophils (IQR), × 10⁹/l	3.3 (2.2–4.2)	4.1 (2.8–5.3)	3.0 (2.2–4.2)	<0.001
Lymphocyte (IQR), × 10⁹/l	1.0 (0.9–1.5)	1.0(0.8–1.4)	1.1 (0.9–1.5)	0.182
Platelets (s.d.), × 10⁹/l^b^	171.7 ± 60.9	171.2 ± 65.3	171.8 ± 60.1	0.933
Interleukin-6 (IQR), pg/ml^c^	4.1 (1.5–7.0)	6.5 (1.5–30.9)	4.4 (1.5–6.5)	0.058
C-reactive protein (IQR), mg/l^d^	18.1 (5.3–38.3)	31.1 (8.4–63.9)	17.4 (5.0–36.7)	0.001
Alanine aminotransferase (IQR), U/l	24.0 (15.5–38.0)	29.0 (17.1–43.6)	23.1 (15.0–37.4)	0.117
Aspartate aminotransferase (IQR), U/l	26.7 (19.0–34.0)	27.2 (20.0–37.0)	26.0 (19.0–34.0)	0.225
Creatinine (IQR), μmol/l	60.0 (48.3–72.4)	62.8 (49.3–75.5)	59.3 (48.0–72.0)	0.855
Troponin I/T > 99th percentile upper reference limit ^e^	45 (11.4%)	12 (19.4%)	33 (9.9%)	0.032
Creatine kinase (IQR), U/l	64.0 (43.0–104.0)	66.0 (40.8–123.5)	63.6 (43.0–104.0)	0.106
Lactate dehydrogenase (IQR), U/l^f^	193.0 (154.0–259.5)	232.0 (162.0–287.0)	189.0 (153.0–250.0)	0.003
NT-Pro-BNP (IQR), pg/ml ^g^	62.0 (24.6–227.0)	152.0 (57.3–1151.4)	50.0 (23.2–193.0)	0.001

IQR, inter-quartile range; NT-Pro-BNP, N-terminal pro-brain natriuretic peptide; s.d., standard deviation.

a Significant difference (*P* < 0.05) was determined between the hypertension and non-hypertension subgroups.

b Platelet count was available in 489 patients.

c Interleukin-6 was available in 145 patients.

d C-reactive protein was available in 492 patients.

e TnI/TnT was available in 394 patients.

f Lactate dehydrogenase was available in 490 patients.

g NT-Pro-BNP available in 131 patients.

### General and anti-hypertensive treatment

Almost all the patients enrolled in this study received various antiviral treatments, and many of them were also under antibiotics and oxygen therapy (99.0%, 71.1% and 48.1%), with no significant difference observed between hypertension and non-hypertension groups. In all the 81 COVID-19 patients with hypertension, 58 (71.6%) of them were taking prescribed medications to lower blood pressure, including 41 (50.6%) received calcium channel blockers, 25 (30.9%) received renin−angiotensin system (RAS) blockers, 14 (17.3%) received *β*-blockers and 8 (9.9%) received diuretics (Supplemental Table 1). However, only 51 (63.0%) of patients treated with anti-hypertensive medicine had achieved control under the goal (<140/90 mmHg).

### Prognosis analysis

Severe COVID-19 occurred in 85 patients (17.1%), including 56 (11.2%) of whom were admitted to the ICU, 28 (5.6%) underwent mechanical ventilation and 10 (2.0%) died. Compared with those without hypertension, severe illness more frequently occurred in patients with pre-existing hypertension (21/81 (25.9%) *vs.* 64/417 (15.3%), *P* = 0.021), mainly contributed by the higher percentage of ICU admission (15/81 (18.5%) *vs.* 41/417 (9.8%), *P* = 0.024). The median hospital stay was 19.6 (18.9–20.4) days, with no difference between hypertension and non-hypertension groups (19.3 (17.3–21.3) days *vs.* 19.8 (19.0–20.7) days, *P* = 0.609).

Though the higher frequency of severe illness occurred in the hypertension group suggested a possible adverse effect, however, after multivariable adjustment, the pre-existence of hypertension was not significantly associated with severe COVID-19 (OR 1.331 (95% CI 0.725–2.442), *P* = 0.356), indicating that the risk of severe COVID-19 was not significantly increased among subjects with pre-existing hypertension compared with those without (Table 3). Data on the risk of COVID-19 prognosis associated with pre-existing hypertension were similar in stratified analyses (Table 4). This result suggested that the findings obtained for the entire cohort were not modified by age groups, sex, type of hospital and blood pressure level on admission. The multivariable logistic regression analysis identified age to be an independent contributor for the severity of COVID-19 (OR 1.029 (95% CI 1.011–1.047); *P* = 0.002).

**Table 3.** Adjusted odd ratios of severe condition in COVID-19 patients

	Univariate analysis		Multivariable analysis	
	OR (95% CI)	*P* value	OR (95% CI)	*P* value^a^
Hypertension (*vs* not present)	2.310 (1.242–4.299)	0.008	1.331 (0.725–2.442)	0.356
Age, years	1.062 (1.040–1.084)	<0.001	1.029 (1.011–1.047)	0.002
Male sex (*vs.* female)	1.062 (0.619–1.824)	0.827	–	–
Diabetes (*vs.* not present)	2.848 (1.350–6.008)	0.006	1.361 (0.640–2.894)	0.423
Coronary heart disease (*vs.* not present)	3.241 (0.815–12.887)	0.095	1.082 (0.254–4.622)	0.915
Chronic kidney disease (*vs.* not present)	3.741 (0.670–20.880)	0.133	–	–

a *P* value was determined by multivariable logistic regression model.

**Table 4.** Comparative adjusted odd ratios of severe condition between the hypertension and non-hypertension group for each subgroup in the overall COVID-19 patients

	Hypertension (*n* = 81)	Non-hypertension (*n* = 417)	OR (95% CI)^a^	Interaction *P* value
Age				0.462
<65	10/52 (19.2%)	52/368 (14.1%)	1.123 (0.443–2.612)	
≥65	11/29 (37.9%)	12/49 (24.5%)	1.683 (0.554–5.067)	
Sex				0.329
Female	12/37 (32.4%)	32/202 (15.8%)	2.043 (0.790–5.133)	
Male	9/44 (20.5%)	32/215 (14.9%)	0.859 (0.306–2.212)	
Type of hospital				0.504
Teaching	10/32 (31.3%)	30/140 (21.4%)	0.977 (0.330–2.750)	
Non-teaching	11/49 (22.4%)	34/277 (12.3%)	1.446 (0.570–0.424)	
Blood pressure on admission				0.628
>140/90 mmHg	7/30 (23.3%)	8/67 (11.9%)	1.641 (0.409–6.299)	
≤140/90 mmHg	14/51 (27.5%)	56/350 (16.0%)	1.367 (0.607–2.939)	

OR, odds radio; CI, confident intervals.

a *P* value was determined by multivariable exact logistic regression model.

High blood pressure (>140/90 mmHg) on admission was also not an independent indicator for bad prognosis in COVID-19 patients (OR 1.108 (95% CI 0.554–2.217), *P* = 0.771), assessed by multivariate logistic regression analysis. We also examined the interaction of RAS blockers use with severe COVID-19 in the hypertension subgroup, in which RAS blockers medication showed no influence on the disease progress (OR 0.739 (95% CI 0.236–2.311), *P* = 0.603).

### Sensitivity analyses

As shown in Supplementary Table 2, the association between severe COVID-19 and hypertension were consistent regardless of whether hypertension was diagnosed through loose or strict criteria. The association was also unchanged in the 1:1 propensity-score matching cohorts (Supplementary Tables 3 and 4).

## Discussion

In this multi-centre observational study in China, we found that COVID-19 patients with hypertension were more likely to develop into severe clinical conditions. However, pre-existing hypertension was not independently associated with a high risk of severe COVID-19. Senior age and underlying cardiovascular-related comorbidities were more commonly observed in hypertension patients, which were all factors predisposing to severe illness and high fatality, probably explaining the high incidence of severe illness in hypertension patients [14].

To date, there is no mechanistic evidence support that a history of hypertension can deteriorate acute infection. The previous studies about other pneumonia demonstrated that pre-existing hypertension could not independently contribute to disease progression [15, 16]. Concurrently, multiple studies on COVID-19 evaluating whether the history of hypertension is a risk factor for adverse outcomes have yielded conflicting results [17–22]. Specifically, Pan *et al*. found that hypertension has an HR of 1.80 for in-hospital mortality in 996 patients with COVID-19 [17], and Gao *et al*. found hypertension has an HR of 2.21 for in-hospital mortality in 2877 patients [18]. In contrast, two other studies that, respectively, enrolled 416 and 1591 patients suggested that after the adjustment for confounders, hypertension was no longer an independent risk factor for COVID-19 [19, 20].

Consistently, the suspected association of severe COVID-19 and hypertension is also not confirmed by our finding. These heterogeneous results may stem from the inconsistency of study populations. In the current cohort study, we enrolled COVID-19 patients in 6 Chinese hospitals remote to the first epicentre of the worldwide pandemic, Wuhan. Thus these patients underwent better management than those from regions suffering from serious healthcare system overload. Besides, distinct from the studies mentioned above, patients involved in our study were generally younger, which may also explain the lower rate of adverse events. Overall, our study could be a meaningful reference for understanding the real role of pre-existing hypertension in COVID-19 progression. However, given the relatively small sample size, our conclusion should be interpreted with caution before any convincing conclusion derived from large-scale studies in ethnically diverse cohorts.

This study provides several additional findings. Firstly, the conclusion that hypertension does not influence COVID-19 severity applies to both sexes, to different age groups, to different levels of hospital, as well as to both patients with high and normal blood pressure. Secondly, hypertension patients more probably suffered from cardiac complications, indicated with the elevation of cardiac biomarkers. However, it is unclear whether these results stem from the high sensitivity to pro-injury factors or the aggravation of pre-existing cardiovascular and metabolic dysfunction. Thirdly, the treatment with RAS blockers was not associated with a higher risk of severe COVID-19, consistent with several recent studies [23, 24]. For cardiovascular patients under regular antihypertensive treatment, it has been suggested that they should discontinue RAS blockers. SARS-CoV-2 enters into the host cell through ACE2 [2]. The RAS blockers may induce the overexpression of ACE2 and subsequently increase the susceptibility of host cells to SARS-Cov-2 invasion [25−27]. The finding from our and other studies could not confirm this concern regarding the potentially harmful effect of RAS inhibitors on COVID-19 severity.

Even though hypertension is not a risk factor for severe COVID-19, more attention and treatment should be offered to patients with underlying hypertension, since they are older, having more comorbidities and more susceptible to cardiac complications. Given that growing evidence has proved that COVID-19 patients under RAS blockers treatment had similar clinical outcomes compared with those not, it is necessary to persist with standard antihypertensive treatment under the COVID-19 pandemic to avoid potential harm caused by uncontrolled hypertension. The treatment and control rates of hypertension in our cohort are higher than those in the general Chinese population but are still unsatisfactory [28].

### Limitations

This study had several limitations. Firstly, given the retrospective nature of this study, some parameters were not available in all patients. For example, we did not monitor dynamic changes in blood pressure during hospitalisation. Instead, we analysed the control rate of hypertension patients relying on blood pressure records on admission. Secondly, we defined underlying hypertension mainly based on the medical record, and we did not take the underestimation of hypertension into account under the COVID-19 emergency. So, the underlying hypertension of some patients might not be diagnosed and was grouped into the non-hypertension cohort. Thirdly, the sample size of this cohort is relatively small and restricted in China, with a low rate of outcome events and comorbid hypertension. Thus, the conclusion should be further confirmed by large-scale prospective cohort studies in ethnically diverse cohorts.

## Conclusions

In this multi-centre observational study involving 498 patients hospitalised due to COVID-19 outside Wuhan city, we confirmed previous observations suggesting that COVID-19 patients with underlying hypertension have a poorer prognosis than those without hypertension. We identified no harmful association of pre-existing hypertension with COVID-19 severity, after adjusted for confounders.

## Data Availability

The datasets used and analysed during the current study are available from the corresponding author on reasonable request.

## Supplementary material

For supplementary material accompanying this paper visit https://doi.org/10.1017/S0950268820003118. click here to view supplementary material
