# Supplementary material for: The influence of pre-existing hypertension on coronavirus disease 2019 patients
Source: Epidemiol Infect. 2021 Jan 5;149:e4. doi: 10.1017/S0950268820003118 (PMC7804074; doi:10.1017/S0950268820003118)
Supplement: Supplementary file 1 [file S0950268820003118sup001.docx]

**Supplemental Table 1. Anti-hypertensive Therapeutics Received by 81 COVID-19 Patients with Hypertension**

| **Anti-hypertensive Therapeutics** | | **No (%)** |
| --- | --- | --- |
| RAS blockers * | | 20 (24.7%) |
|  | Valsartan | 9 |
|  | Irbesartan | 5 |
|  | Telmisartan | 3 |
|  | Perindopril | 2 |
|  | Olmesartan | 1 |
|  | losartan | 1 |
| β-blockers | | 14 (17.3%) |
|  | Metoprolol | 10 |
|  | Bisoprolol | 4 |
| Calcium channel blockers | | 41 (50.6%) |
|  | Amlodipine | 19 |
|  | Nifedipine | 17 |
|  | Felodipine | 5 |
| Diuretics | | 8 (9.9%) |
|  | Furosemide | 6 |
|  | Hydrochlorothiazide | 2 |
| RAS blockers & Diuretics | | 3 (3.7%) |
|  | Irbesartan & Hydrochlorothiazide | 3 |
| RAS blockers & Calcium channel blockers | | 2 (2.5%) |
|  | Valsartan & Amlodipine | 2 |
| None anti-hypertensive therapeutics | | 23 (28.4%) |

Abbreviation: RAS, renin-angiotensin system.

*One patient received both Valsartan and Telmisartan.

**Supplementary Table 2. Risk of Severe Condition Associated with Hypertension in COVID-19 Patients**

| **Outcome** | **No (%)** | ***P* value^a^** | **OR (95% CI)** **^b^** | ***P* value^c^** |
| --- | --- | --- | --- | --- |
| Hypertension^d^ | 29/148 (19.6%) | 0.330 | 0.950 (0.556-1.621) | 0.850 |
| Non-hypertension | 56/350 (16.0%) |  | Reference |  |

^a^ P value was determined by Chi-square or Fisher’s exact test; ^b^ Adjusted for age, sex, diabetes, coronary heart disease and kidney disease; ^c^ P value was determined by multivariable logistic regression model; ^d^ Hypertension was diagnosed through a loose criterion: Patients with a clear record of preexisting hypertension or showed a blood pressure over 140/90 mmHg on admission were diagnosed as hypertension.

**Supplementary Table 3. Baseline Characteristics of Patients with and without Hypertension before and after Matching**

|  | **Un-matched patients** | | | **Matched patients** | | |  |
| --- | --- | --- | --- | --- | --- | --- | --- |
|  | **Hypertension (n=81)** | **Non-hypertension (n=417)** | ***P* value^a^** | **Hypertension (n=68)** | **Non-hypertension (n=68)** | ***P* value^a^** | **SMD (%)** |
| Age (SD), years | 59.1 (56.2-62.0) | 46.9 (45.5-48.3) | <0.001 | 56.6 (53.8-59.5) | 56.4 (53.3-59.4) | 0.888 | 2.260 |
| Female sex | 37 (45.7%) | 202 (48.2%) | 0.716 | 30 (44.1%) | 31 (45.6%) | 0.863 | 2.934 |
| Hospital level | | | | | | | |
| Teaching | 32 (39.5%) | 140 (33.6%) | 0.304 | 24 (35.3%) | 25 (36.8%) | 0.858 | -2.990 |
| Non-teaching | 49 (60.5%) | 277 (66.4%) |  | 44 (64.7%) | 43 (63.2%) |  |  |
| Diabetes | 19 (23.5%) | 24 (5.8%) | <0.001 | 13 (19.1%) | 13 (19.1%) | >0.999 | 0.000 |
| Hyperlipidemia | 8 (9.9%) | 6 (1.4%) | <0.001 | 4 (5.9%) | 2 (2.9%) | 0.680 | 9.797 |
| Chronic obstructive pulmonary disease | 3 (3.7%) | 2 (0.5%) | 0.032 | 1 (1.5%) | 2 (2.9%) | >0.999 | 7.739 |
| Chronic kidney disease | 4 (4.9%) | 2 (0.5%) | 0.008 | 0 (0.0%) | 1 (1.5%) | >0.999 | 6.745 |
| Coronary heart disease | 4 (4.9%) | 6 (1.4%) | 0.063 | 2 (2.9%) | 4 (5.9%) | 0.680 | 7.350 |
| Distance | - | | | 0.232 (0.197-0.266) | 0.229 (0.195-0.263) | 0.922 | 1.470 |

Abbreviation: SD, standard deviation; SMD, standardized mean difference;

^a^ P value was determined by Chi-square or Fisher’s exact test

**Supplementary Table 4. Outcomes in Un-matched and Matched Populations**

|  | **Unmatched patients** | | | **Matched patients** | | | | |
| --- | --- | --- | --- | --- | --- | --- | --- | --- |
|  | **Hypertension** | **Non-hypertension** | ***P* value** ^a^ | **Hypertension** | **Non-hypertension** | ***P* value** ^a^ | **OR (95% CI)** **^b^** | ***P* value^c^** |
|  | **n=81** | **n=417** |  | **n=68** | **n=68** |  |  |  |
| Composite endpoint | 21 (25.9%) | 64 (15.3%) | 0.021 | 13 (19.1%) | 18 (26.5%) | 0.307 | 1.600  (0.697-3.673) | 0.268 |
| ICU admission | 15 (18.5%) | 41 (9.8%) | 0.024 | 7 (10.3%) | 14 (20.6%) | 0.097 |  |  |
| Ventilation | 4 (4.9%) | 24 (5.8%) | >0.999 | 4 (5.9%) | 4 (5.9%) | >0.999 |  |  |
| Death | 4 (4.9%) | 6 (1.4%) | 0.063 | 3 (4.4%) | 3 (4.4%) | >0.999 |  |  |

Abbreviation: OR, odds radio; CI, confident intervals

^a^ P value was determined by Chi-square or Fisher’s exact test; ^b^ Adjusted for age, sex, diabetes, coronary heart disease and kidney disease; ^c^ P value was determined by multivariable logistic regression model
